# Redox signals and oxidative stress in the control of mitochondrial protein import

**DOI:** 10.1002/pro.70665

**Published:** 2026-06-02

**Authors:** Lidwina Hasberg, Viktoria Katharina Lauterbach, Torsten Ochsenreiter, Jan Riemer

**Affiliations:** ^1^ Redox Metabolism Group, Institute for Biochemistry, Cologne Excellence Cluster on Cellular Stress Responses in Aging‐Associated Diseases (CECAD), and Center for Molecular Medicine (CMMC) University of Cologne Cologne Germany; ^2^ Institute of Cell Biology University of Bern Bern Switzerland

**Keywords:** disulfide relay, mitochondrial protein import, oxidative stress, reactive oxygen species (ROS), redox signaling

## Abstract

Mitochondrial protein import is essential for organelle biogenesis and cellular homeostasis. It operates in an environment that is intrinsically shaped by redox chemistry. Mitochondria are major sources of reactive oxygen species (ROS), which arise as by‐products of oxidative phosphorylation. Cells therefore maintain sophisticated ROS‐handling systems, including compartmentalized antioxidant networks, to balance redox signaling with protection from oxidative stress. Increasing evidence indicates that these redox conditions directly influence mitochondrial protein import at multiple levels. In this review, we provide an overview of ROS production, ROS signaling, and oxidative stress in relation to mitochondrial protein import. We outline the major mitochondrial protein import pathways, and discuss how their activity is modulated by redox‐dependent mechanisms. A particular focus is placed on the mitochondrial disulfide relay system of the intermembrane space, which directly couples protein import to redox chemistry through oxidative folding, and how it is influenced by the local redox environment. Collectively, we propose that mitochondrial protein import is partially governed by redox‐dependent mechanisms, enabling integration of metabolic state, stress responses, and signaling pathways.

## 1 INTRODUCTION

Mitochondrial protein import ensures the accurate delivery, folding, and assembly of hundreds of nuclear‐encoded proteins into distinct mitochondrial subcompartments. While it is traditionally seen as a constitutive housekeeping function of the cell, recent studies demonstrated that mitochondrial protein import is tightly linked to cellular signaling networks including the dynamic regulation by cellular redox conditions. Mitochondria are among the primary intracellular sources of reactive oxygen species (ROS). In particular, hydrogen peroxide (H_2_O_2_) has emerged as a key signaling molecule that modulates protein function through reversible oxidative modifications, while excessive ROS levels lead to oxidative stress and proteotoxic damage. Given that mitochondrial proteins must be imported in a largely unfolded and redox‐sensitive state, oxidative conditions can directly influence multiple steps of the import process, from precursor stability and translocation efficiency to folding and quality control. Thus, mitochondrial protein import operates at the interface of metabolism and redox homeostasis, making it critical for cellular adaptation to fluctuating physiological and stress conditions.

## 2 ROS AND THE FINETUNED BALANCE OF OXIDATIVE STRESS AND REDOX SIGNALING

ROS are small reactive molecules originally derived from molecular oxygen (Sies et al., 2022; Sies & Jones, 2020; Winterbourn, 2008). Major cellular ROS include superoxide anions (O_2_^•−^), H_2_O_2_, and hydroxyl radicals (^•^OH), which markedly differ in their reactivity, lifetime, and biological function (Murphy et al., 2011; Winterbourn, 2008). While excessive ROS accumulation causes oxidative damage of biomolecules during conditions of oxidative stress, a tightly controlled ROS production is indispensable for physiological signaling (Forman & Zhang, 2021; Sies & Jones, 2020; Travasso et al., 2017). The balance between deleterious oxidative stress and beneficial redox signaling is therefore a central determinant of cellular homeostasis.

**FIGURE 1:**
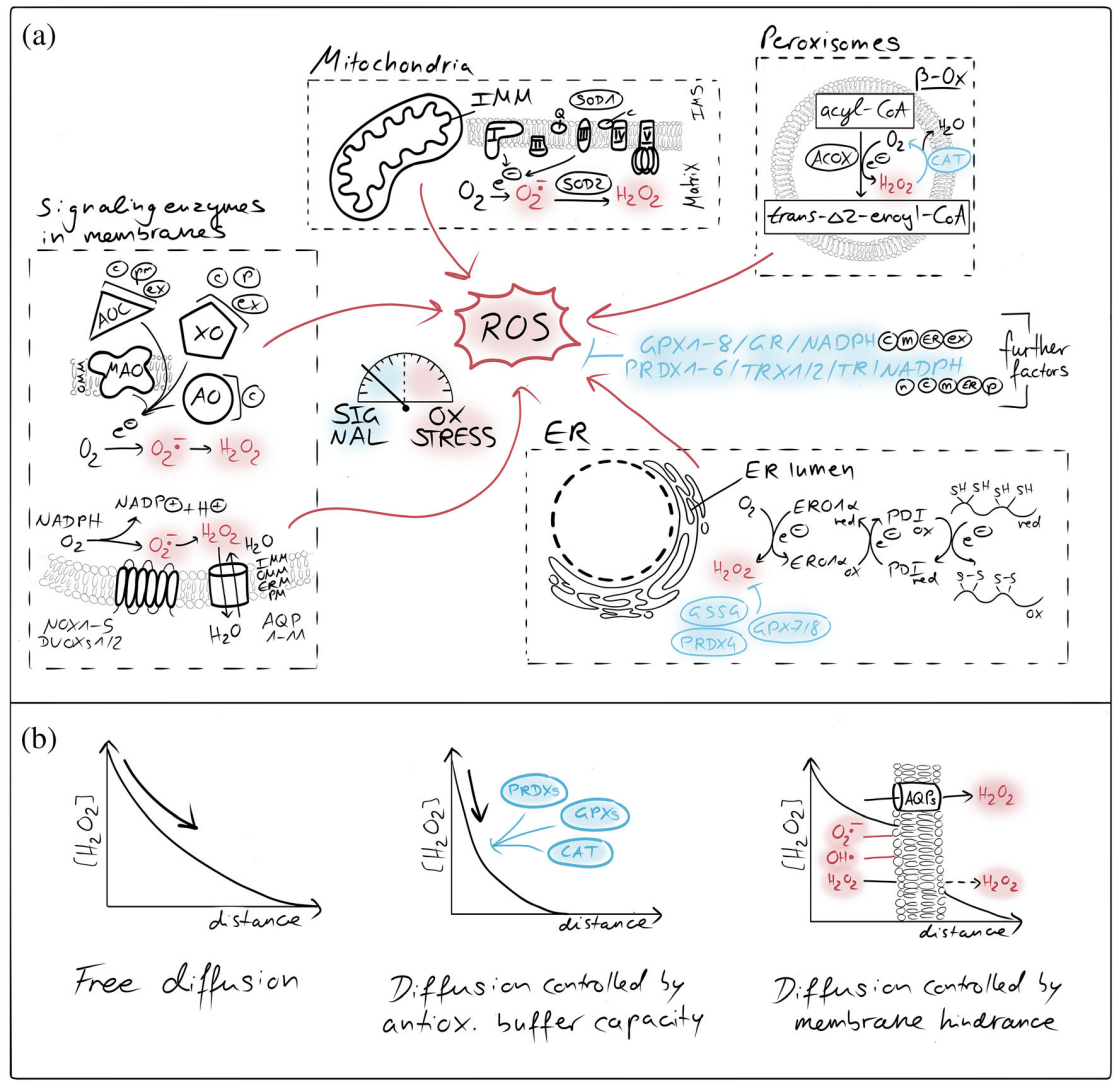
Compartmentalized ROS generators and antioxidant systems. (a) Cellular sources and detoxification pathways of reactive oxygen species (ROS). Major intracellular ROS generation sites include the mitochondrial respiratory chain, peroxisomes, endoplasmic reticulum (ER), and membrane‐associated NADPH oxidases (NOXs). Superoxide (O_2_^•−^) and hydrogen peroxide (H_2_O_2_) produced in these compartments are spatially confined and tightly controlled by compartment‐specific antioxidant systems. Key detoxification pathways include superoxide dismutases (SODs), peroxiredoxins (PRDXs), glutathione peroxidases (GPXs), catalase (CAT), and the glutathione/thioredoxin systems, which collectively maintain redox homeostasis by suppressing oxidative stress while permitting localized ROS signaling. AO, aldehyde oxidase; MAO‐A/B, monoamine oxidase type A/B; AOC, (di)amine oxidase; XO/XDH, xanthine oxidase/dehydrogenase; AQP, aquaporins; NOX/DUOX, NADPH oxidases/dual oxidases; ACOX, acyl‐CoA oxidase; c, cytosol; m, mitochondria; ER, endoplasmic reticulum; p, peroxisome; ex, extracellular space; n, nucleus. (b) Membrane barriers and antioxidant systems shape H_2_O_2_ diffusion and signaling. Cellular membranes and compartment‐specific antioxidant systems control the spread of hydrogen peroxide (H_2_O_2_). Biological membranes restrict free diffusion of H_2_O_2_, generating local gradients between organelles and the cytosol. High‐capacity scavenging systems, including peroxiredoxins, glutathione peroxidases, catalase, and the glutathione/thioredoxin networks, act as kinetic sinks that further steepen gradients, confining H_2_O_2_ signals in space and time and preventing oxidative damage.

Mitochondria represent major sites of intracellular ROS production, predominantly through electron leakage from the respiratory chain, particularly at complexes I and III (Brand, 2020; Mailloux, 2021; Murphy, 2009; Zhang & Wong, 2021) (Figure 1a). O_2_^•−^ generated at these sites is rapidly converted into H_2_O_2_ by superoxide dismutases (Wang et al., 2018), with H_2_O_2_ as the principal redox signaling molecule that can be released from mitochondria into the cytosol due to its chemical stability and membrane permeability (Hoehne et al., 2022; Murphy, 2009; Winterbourn, 2008). Additional intracellular ROS sources include NADPH oxidases (NOX) at cellular membranes, disulfide‐forming enzymes in the endoplasmic reticulum, and metabolic enzymes such as various dehydrogenases of the *β*‐oxidation in peroxisomes, xanthine oxidases, aldehyde oxidases, and monoamine oxidases as well as non‐enzymatic ROS generation (Appenzeller‐Herzog et al., 2016; Ferko et al., 2024; Fransen & Lismont, 2019; Gupta & Singh, 2025; Roscoe & Sevier, 2020; Schroder, 2020; Walker et al., 2018) (Figure 1a). The distinct distribution of ROS generators throughout the cell and the fluctuating flux of electrons through these systems thereby allow a spatially and temporally tightly controlled ROS production across cellular (sub)compartments.

Antioxidant systems are essential for the detoxification of ROS and are present in most cellular (sub)compartments (Figure 1a). These systems include peroxiredoxins, glutathione peroxidases, and catalases, all of which finally depend on the sufficient supply of NADPH to sustain the reductive capacity (Flohe et al., 2022; Gebicka & Krych‐Madej, 2019; Gencheva & Arnér, 2022; Glorieux & Buc Calderon, 2024; Hasan et al., 2022; Villar et al., 2023; Winterbourn, 2025). Together, these systems form an efficient antioxidative network that rapidly scavenges ROS and prevents uncontrolled oxidative damage upon ROS accumulation. A consequence of this antioxidative activity is the temporal and spatial restriction of ROS. Both antioxidative enzymes and the permeability of cellular membranes substantially limit the diffusion of ROS and thereby constrain the distance over which ROS acts (Fisher, 2009; Mishina et al., 2019; Moller et al., 2019; Nordzieke & Medraño‐Fernandez, 2018) (Figure 1b). For H_2_O_2_, quantitative measurements demonstrated the existence of steep concentration gradients from mitochondria with small to no amounts of H_2_O_2_ detected in the cytosol (Hoehne et al., 2022; Koren et al., 2023; Pak et al., 2020; van Soest et al., 2024). These observations indicate that H_2_O_2_ concentrations sufficient for redox signaling are mainly achieved in close proximity to sites of ROS generation. These can happen for example close to the respiratory chain in matrix, intermembrane space (IMS) and at the mitochondrial surface, or within spatially confined microdomains without antioxidative capacity like presumably at organelle contact sites (Beretta et al., 2020; Booth et al., 2016; Sorrentino et al., 2022; Woo et al., 2010). Although H_2_O_2_ is membrane permeable, its passive diffusion across membranes appears too slow and inefficient for rapid redox signaling between cellular (sub)compartments. This is underscored by the requirement for H_2_O_2_‐permeable aquaporins in NOX‐dependent redox signaling pathways (Miller et al., 2010; Nordzieke & Medraño‐Fernandez, 2018). These findings support the concept that ROS generation and ROS signaling largely occur within the same cellular (sub)compartment. Notably, the mitochondrial surface seems to be an exception, as porins in the mitochondrial outer membrane (OMM) allow for unrestricted diffusion of H_2_O_2_, potentially enabling the redox communication between the IMS and the cytosol. In summary, the compartmentalization of ROS‐generating systems together with antioxidative systems establishes discrete redox environments within the cytosol and mitochondrial subcompartments (Herb et al., 2021).

Oxidative stress occurs if excessive amounts of ROS overwhelm the cellular antioxidative and repair capacity (Forman & Zhang, 2021; Sies & Jones, 2020; Travasso et al., 2017). This redox imbalance can result from mitochondrial dysfunction, impaired antioxidative systems, dysfunctional ROS production enzymes, chronic inflammation, or environmental insults. Oxidative damage due to ROS affects all classes of biomolecules, leading to protein carbonylation, cysteine and methionine oxidation, lipid peroxidation, and mutagenic DNA damage (Le Boulch et al., 2018; Linden et al., 2008; Mukherjee et al., 2020; Nakamura et al., 2003; Navarro‐Ruiz et al., 2022; Valverde et al., 2018). Cells are equipped with dedicated repair systems to counteract this oxidative damage. For example, oxidized amino acid residues in proteins can be repaired by sulfiredoxins or methionine sulfoxide reductases. Oxidative DNA lesions are for example corrected by base excision repair pathways, while lipid peroxidation products are detoxified by glutathione‐dependent enzymes, including glutathione peroxidases, and damaged membranes are restored through phospholipid remodeling pathways and organelle‐selective turnover. Importantly, oxidative stress is not merely defined by elevated ROS levels but rather by a combined failure of the redox buffer capacity and repair fidelity (Forman & Zhang, 2021). Thus, it is not surprising that oxidative damage is implicated in aging and a broad spectrum of diseases, including neurodegeneration, cancer, cardiovascular disorders, and metabolic syndromes (Forman & Zhang, 2021; Ghosh & Shcherbik, 2020; Guo et al., 2013; Sies & Jones, 2020; Xiao et al., 2020; Xu et al., 2025).

In contrast, redox signaling relies on local reversible protein oxidation events, modulating the protein function and stability without damage (Forman et al., 2014; Woo et al., 2010; Yamamoto et al., 2018). In this process, the selective oxidation of cysteine residues within the target protein, leading to reversible modifications such as sulfenylation, glutathionylation, or disulfide bond formation, is of high importance. These redox switches regulate various processes, including kinase and phosphatase activities, transcription factor (de)activation, metabolic fluxes, and organelle dynamics (Ahn & Thiele, 2003; Anastasiou et al., 2011; Dansen et al., 2009; Dayalan Naidu et al., 2018; Echtay et al., 2002; Hao et al., 2006; Jin et al., 2011; Kim et al., 2018; Leslie et al., 2003; Li et al., 2007; Manalo et al., 2002; Meng et al., 2002; Nanadikar et al., 2023; Peralta et al., 2015; Reynaert et al., 2006; Shutt et al., 2012; van der Reest et al., 2018; Welsh & Madan, 2024; Yamamoto et al., 2008; Zhang et al., 2017). In general, H_2_O_2_ serves as a key second messenger, enabling rapid and reversible signal propagation in a selective manner through restricted diffusion and tight enzymatic control. In this context, antioxidants, particularly peroxiredoxins, are crucial for shaping and relaying redox signals within the cell (Delaunay et al., 2002; Travasso et al., 2017; Veal & Kritsiligkou, 2024).

Collectively, oxidative stress and redox signaling represent two sides of the same “biochemical coin,” indicating ROS to have broad pleiotropic as well as very specific effects on diverse cellular processes, among others mitochondrial protein import.

## 3 THE DIVERSITY OF MITOCHONDRIAL PROTEIN IMPORT PATHWAYS

**FIGURE 2:**
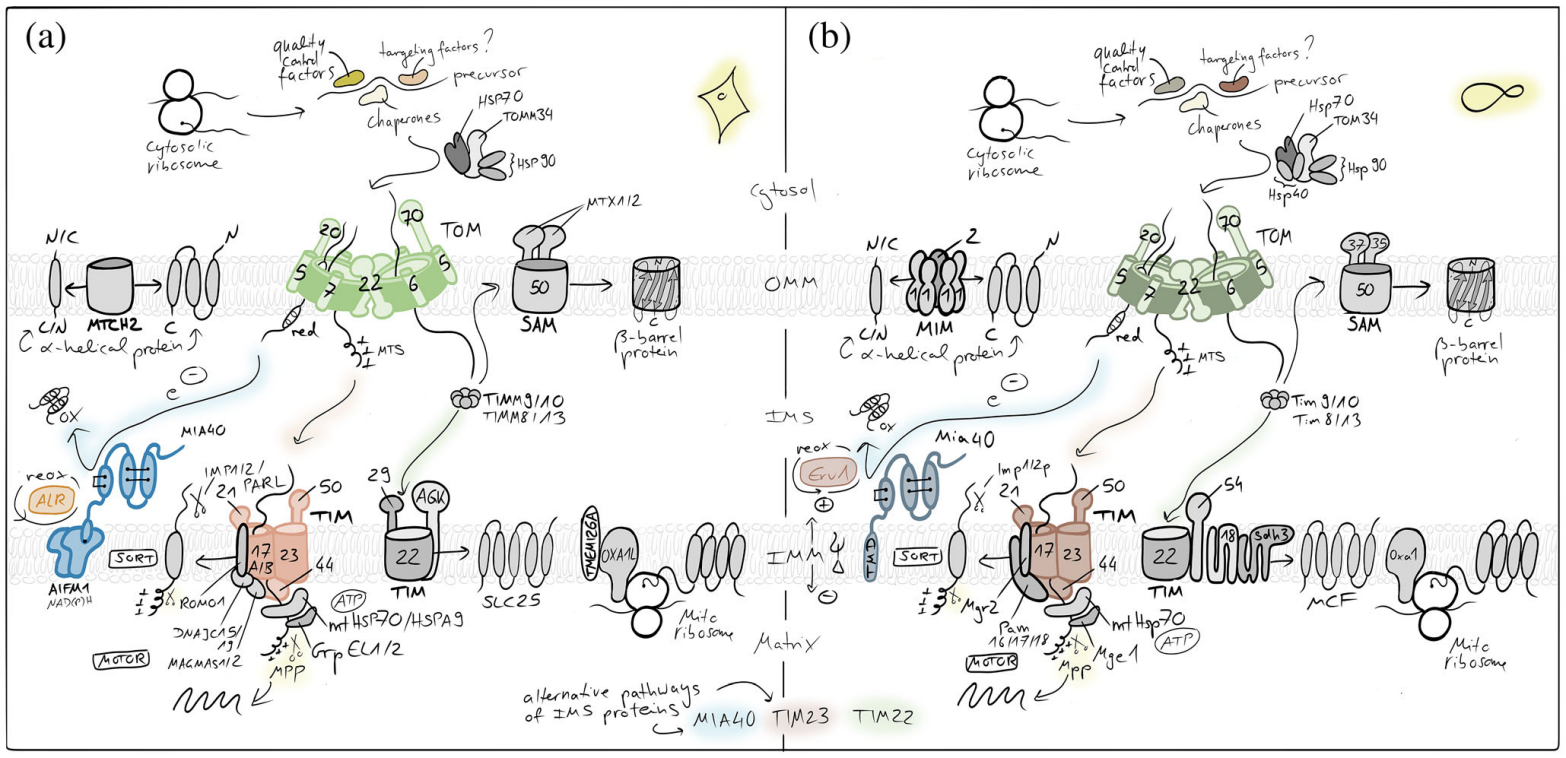
Mitochondrial protein import pathways. Most nuclear‐encoded proteins are synthesized in the cytosol and imported via the TOM complex in the outer membrane (OMM), followed by sorting through distinct inner membrane (IMM) and matrix pathways. Presequence‐containing proteins are translocated by the TIM23 machinery into the matrix or the IMM, while hydrophobic carrier proteins (members of the SLC25 family) use the TIM22 pathway. Intermembrane space (IMS) proteins are imported and oxidatively folded by the disulfide relay system, *β*‐barrel OMM proteins are assembled by the SAM complex and *α*‐helical OMM proteins utilize the MIM/MTCH2 pathways. OXA1 supports the co‐translational insertion of mitochondria‐encoded proteins into the IMM and has been implied in IMM‐insertion of select nuclear‐encoded proteins from the matrix side. Together, these pathways ensure accurate targeting and maturation of the mitochondrial proteome.

Mammalian mitochondria contain more than one thousand proteins, of which the majority are encoded in the nuclear genome and synthesized on cytosolic ribosomes. Therefore, the efficient and accurate import of these precursor proteins into mitochondria is essential for organelle biogenesis, metabolic function, and cellular homeostasis. To localize the diversity of mitochondrial proteins to their distinct mitochondrial subcompartments, eukaryotic cells evolved multiple and specific protein import pathways within mitochondria (Endo & Wiedemann, 2025; Ghifari et al., 2025; Jain et al., 2025; Lee‐Glover & Shutt, 2024; Leeming et al., 2026; Zarges & Riemer, 2024) (Figure 2).

Protein import into mitochondria begins at cytosolic ribosomes from where precursor proteins have to reach the translocase of the outer membrane (TOM) complex, which serves as the entry gate for nearly all precursor proteins (den Brave et al., 2024; Endo & Wiedemann, 2025; Nussberger et al., 2024; Ozdemir & Dennerlein, 2024). Cytosolic chaperones maintain the precursor proteins in an import‐competent, unfolded state and guide them to the surface receptors of the TOM complex, primarily TOMM20, TOMM22, and TOMM70, for translocation (Balzarini et al., 2025; Endo & Wiedemann, 2025; Felipe Perez et al., 2024; Ghifari et al., 2025; Lee‐Glover & Shutt, 2024; Leeming et al., 2026). The ubiquitin proteasome system plays an important role in surveying the fidelity of mitochondrial protein import by in some instances degrading the majority of precursors *en route* to the mitochondrial surface (Eldeeb et al., 2020; Finger et al., 2020; Kim et al., 2023; Lenhard et al., 2023; McMinimy et al., 2024; Phu et al., 2020; Rodl & Herrmann, 2023; Salscheider et al., 2022; Sladowska et al., 2021; Weith et al., 2025). From the TOM pore, the precursor proteins are sorted for distinct downstream pathways based on their targeting signals embedded within their primary sequence or structural features to transport them to the different subcompartments of mitochondria.

Proteins destined for the mitochondrial matrix or inner membrane (IMM) that possess an N‐terminal, positively charged amphipathic helix as a mitochondrial targeting signal (MTS) are transported through the presequence translocase of the IMM (TIM) complex, TIM23 (Jain et al., 2025). The translocation across the IMM is driven by the membrane potential (Δψ) and by an ATP‐dependent import motor associated with the TIM23 complex. In the matrix, the presequences are proteolytically removed by mitochondrial processing peptidase, and the proteins fold into their mature conformations. If the N‐terminal presequence is followed by a hydrophobic sorting signal (bipartite MTS), TIM23 mediates the lateral insertion of the protein into the IMM in a “stop‐transfer” mechanism.

Multispanning IMM proteins without cleavable presequences are typically imported via the TIM22 (or “carrier”) pathway (Borrero‐Landazabal et al., 2025; Endo & Wiedemann, 2025; Kizmaz et al., 2024). These hydrophobic proteins, including the metabolite carriers of the SLC25 family, are recognized in the cytosol by TOMM70 and transferred through the TOM complex to small TIMM chaperones like TIMM9/TIMM10 in the IMS. The delivery via the TIM22 complex enables the membrane potential–driven insertion into the IMM, ensuring correct topology and assembly.

A distinct pathway operates for *β*‐barrel proteins of the OMM, such as porins and TOMM40 (Dimogkioka & Rapaport, 2025; Ganesan et al., 2024; Ravi et al., 2025). After translocation through the TOM complex, these precursors are escorted by small TIMM chaperones to the sorting and assembly machinery (SAM) complex, which catalyzes their folding and insertion into the OMM. For *α*‐helical OMM protein insertion into the OMM, the MIM pathway operates in yeast– often in cooperation with TOM, and MTCH2 operates in mammalian cells (Becker et al., 2011; Guna et al., 2022; Kruger et al., 2017; Muthukumar et al., 2024).

IMS proteins follow specialized import routes dependent on their structural folding requirements (Dickson‐Murray et al., 2021; Eaglesfield & Tokatlidis, 2021; Endo & Wiedemann, 2025; Haastrup et al., 2023; Kizmaz et al., 2024; Weith et al., 2025; Zarges & Riemer, 2024). A portion of IMS proteins is imported via stop‐transfer mechanisms through the TIM23 complex, while others rely on the mitochondrial disulfide relay system. In this oxidative protein folding and import pathway, cysteine‐containing precursors are imported through the TOM complex and are subsequently oxidized by the IMS‐localized oxidoreductase MIA40 (mitochondrial intermembrane space import and assembly protein 40, in mammalian cells often called coiled‐coil–helix–coiled‐coil–helix domain‐containing protein 4, CHCHD4), whereby the electrons are transferred to the sulfhydryl oxidase augmenter of liver regeneration (ALR). The disulfide bond formation traps these proteins in the IMS and promotes their maturation and stability.

Proteins encoded by the mitochondrial genome are synthesized on mitochondrial ribosomes within the matrix and co‐translationally inserted into the IMM by dedicated insertases (Antolinez‐Fernandez et al., 2024; Ghifari et al., 2025; Kremer & Rehling, 2024). Together with the protein import pathways of nuclear‐encoded proteins, this system ensures the coordinated assembly of respiratory chain complexes and other mitochondrial protein machineries.

In summary, the mitochondrial protein import is orchestrated by multiple, specialized pathways that rely on targeting signals, the membrane potential, ATP hydrolysis, and the redox environment. The fidelity of these processes is crucial for mitochondrial functionality, and the dysregulation is increasingly recognized as a key contributor to human disease.

## 4 THE IMPACT OF REDOX SIGNALING AND OXIDATIVE STRESS ON MITOCHONDRIAL PROTEIN IMPORT

Early studies already demonstrated that oxidizing conditions affect mitochondrial protein import. Depending on the context, different oxidizing or reducing redox conditions were shown either to impair protein import, leading to the accumulation of unprocessed precursor proteins in the cytosol and reduced levels of mature mitochondrial proteins (Pandey et al., 2006; Wright et al., 1997; Wright et al., 2001) or, conversely, promoting the selective accumulation of specific mitochondrial proteins, including antioxidative enzymes such as superoxide dismutases 1 and 2 (SOD1 and SOD2) (Araujo et al., 2011; Kawamata & Manfredi, 2008; Suzuki et al., 2013). Subsequent work showed that imbalanced redox conditions influence mitochondrial protein import at multiple stages, including precursor synthesis and stability, targeting to mitochondria, translocation efficiency across the OMM and IMM, proteolytic processes, (oxidative) protein folding, and protein quality control. Importantly, redox‐dependent effects on mitochondrial protein import are not exclusively detrimental or irreversible. Instead, redox switches allow adaptive changes, causing mitochondria to dynamically adjust their proteome and protein import capacity in response to oxidative challenges and changes in the metabolic environment.

### 4.1 In the cytosol

**FIGURE 3:**
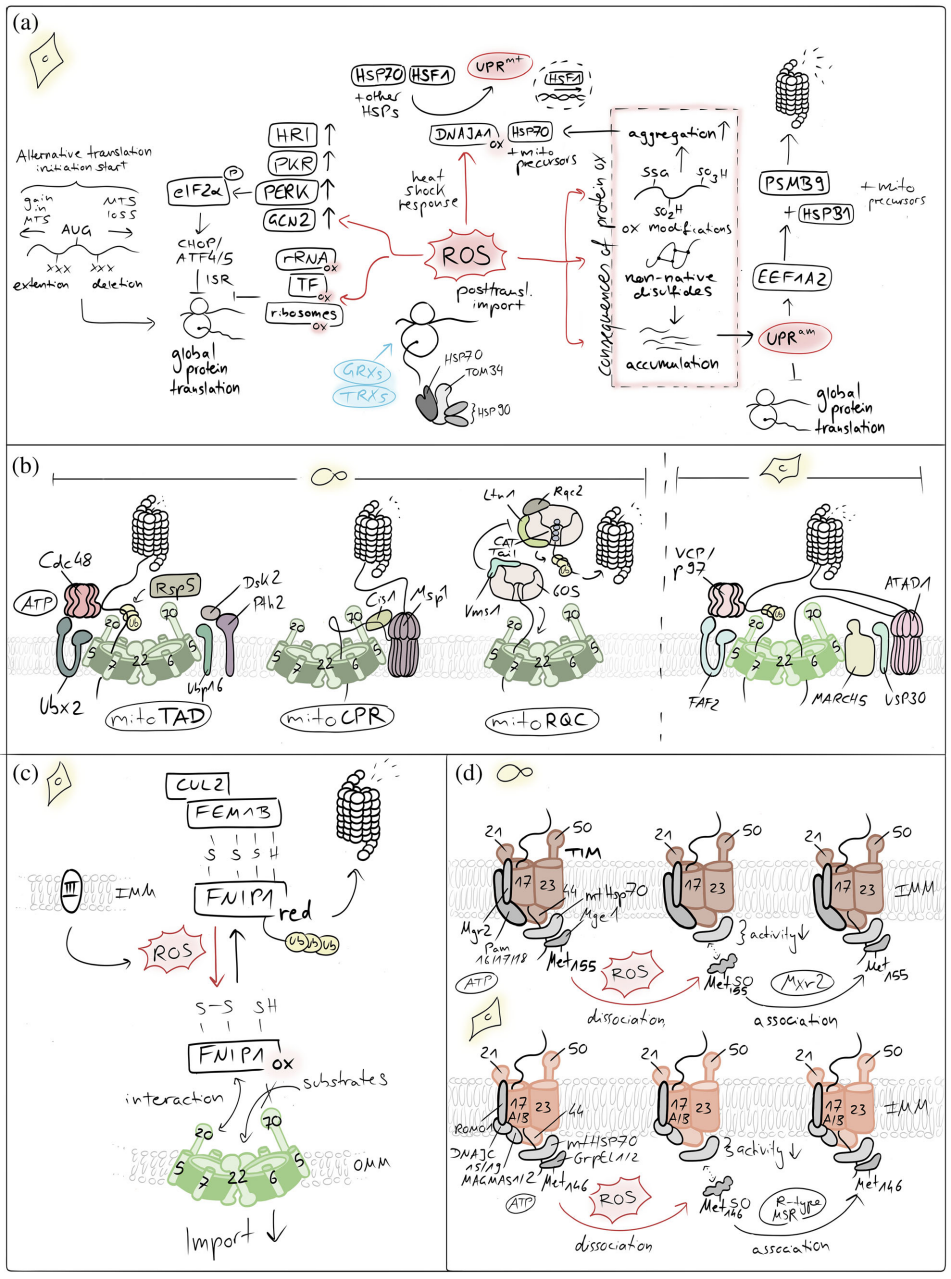
Redox influences on mitochondrial import. (a) ROS‐mediated control of cytosolic translation and proteostasis. Elevated reactive oxygen species (ROS) influence cellular proteostasis. ROS can directly inhibit cytosolic translation and activate stress‐responsive kinases, reducing the influx of newly synthesized proteins into mitochondria. In parallel, mitochondrial ROS trigger the mitochondrial unfolded protein response (UPR^mt^) and the unfolded protein response activated by mistargeting of proteins (UPR^am^), enhancing chaperone expression and proteasomal capacity. If these adaptive pathways are overwhelmed, unimported mitochondrial precursors accumulate in the cytosol, leading to protein aggregation and proteotoxic stress. (b) Quality control pathways that unclog the TOM import channel. Conserved mechanisms resolve stalled mitochondrial protein import at the TOM complex. In yeast, mitoTAD and mitoCPR pathways sense and extract jammed precursor proteins from the TOM channel, promoting their ubiquitination and proteasomal degradation to restore import competence. In mammalian cells, the outer membrane E3 ubiquitin ligase MARCH5 ubiquitinates arrested import intermediates and associated TOM components, marking them for extraction and clearance. This process is counterbalanced by the deubiquitinase USP30, which removes ubiquitin chains from TOM substrates and import intermediates, thereby fine‐tuning the efficiency and reversibility of the unclogging response. (c) Reductive stress sensing controls mitochondrial protein import at the TOM pore. A reductive stress–responsive quality control pathway regulates mitochondrial protein import. Under conditions of elevated cytosolic reducing power, stalled or misfolded precursor proteins accumulate at the TOM complex. The adaptor protein FNIP1 senses this imbalance and recruits the FEMB1–Cullin‐2 (CUL2) E3 ubiquitin ligase complex to the OMM. This promotes ubiquitination and proteasomal clearance of arrested import intermediates at the TOM pore, thereby relieving import congestion and restoring mitochondrial proteostasis. (d) ROS‐dependent oxidation of Mge1 inhibits mitochondrial protein import. Elevated ROS impair mitochondrial protein import by oxidizing methionine residues in the mitochondrial nucleotide exchange factor Mge1. Methionine oxidation disrupts the interaction of Mge1 with the Hsp70 import motor, reducing ATP‐driven cycling of the chaperone and thereby slowing translocation of precursor proteins into the matrix. This redox‐sensitive modification provides a rapid mechanism to couple mitochondrial import activity to oxidative stress.

In general, oxidative disturbances affect cytosolic protein translation by disruption of the translation initiation and elongation processes (Figure 3a). On the one hand, ROS directly causes oxidative damage of ribosomal RNA (rRNA) and proteins (Bollineni et al., 2014; Samluk et al., 2019; Shcherbik & Pestov, 2019; Topf et al., 2018; Willi et al., 2018), leading to ribosomal dysfunction and reduced protein translation efficiency. This fact might particularly affect those mitochondrial proteins that are co‐translationally translocated via the TOM complex into the organelle as at this point the cytosolic ribosomes need to be particularly close to the mitochondrial surface where ROS concentrations have been shown to be higher than in the cytosol (Hoehne et al., 2022; Koren et al., 2023). On the other hand, ROS also directly oxidize and thereby inactivate translation initiation factors. Additionally, oxidative stress triggers the activation of stress kinases, such as protein kinase RNA‐like ER kinase (PERK) and General Control Nonderepressible 2 (GCN2), which phosphorylate and thereby inhibit the eukaryotic translation initiation factor 2*α* (eIF2*α*), leading to a global reduction in protein synthesis including mitochondrial proteins (Amiri et al., 2025; Ghosh & Shcherbik, 2020; Grant, 2011; Shenton et al., 2006).

Redox signals can also modulate translation beyond general attenuation, for example, they can modulate the speed of elongation to allow a slower and efficient production of selenocysteine‐containing antioxidant proteins (Rehfeld et al., 2025), or control the selection of initiation sites during translation which can result in the acquisition or loss of a mitochondrial targeting information (Gerashchenko et al., 2012; Ly et al., 2025). A prominent example is yeast‐Gpx3, normally a cytosolic protein. Redox signals can induce translation start at an upstream start that results in an N‐terminal extension that serves as an MTS driving the protein to the IMS where it serves an antioxidative role (Gerashchenko et al., 2012; Kritsiligkou et al., 2017). The process is independent of the disulfide relay import pathway, though IMS‐Gpx3 has been shown to interact with Mia40 both in vitro and *in organello*. Whether the latter mechanism of proteome plasticity has a broader relevance and also occur in human cells remains poorly understood.

The majority of mitochondrial proteins is imported in a posttranslational manner (Luo et al., 2025; Zhu et al., 2025). The respective precursor proteins are thus sensitive to oxidation prior to import, particularly those containing cysteine residues or metal‐binding motifs (Munakata et al., 2004) Oxidative stress in the cytosol can lead to precursor misfolding or aggregation, for example, through the introduction of non‐native disulfide bonds or amino acid residue modifications. This can render precursor proteins import‐incompetent and trigger their proteasomal degradation (Muller & Hoppe, 2024; Sladowska et al., 2021; Swatek & Komander, 2016; Wrobel et al., 2015). Cytosolic redox systems, including thioredoxins and the glutathione system, therefore indirectly support mitochondrial biogenesis by maintaining precursor proteins in a reduced, import‐competent state (Banci et al., 2013; Durigon et al., 2012; Morgan et al., 2009). This is in particular true for the cysteine‐rich proteins of the IMS, where many of them form disulfide bonds in the mature protein. Additionally, oxidation of cysteines positioned in MTSs or transmembrane regions to sulfinic or sulfonic acid or modification with glutathione can change their biophysical properties by introducing negative charges that might inactivate the MTS or prevent membrane insertion, respectively (Garrido Ruiz et al., 2022; Reina et al., 2020).

The accumulation of misfolded precursors in the cytosol activates the unfolded protein response activated by the mistargeting of proteins (UPR^am^) both in yeast and human cells (Kim et al., 2023; Wrobel et al., 2015) (Figure 3a). This pathway essentially activates the proteasome and leads to lowered cytosolic translation rates, resulting in the inhibition of protein synthesis. This is likely achieved through the activation of elongation factor 1 alpha 2 gene (EEF1A2), which in turn upregulates the proteasome subunit *β* type‐9 (PSMB9) and the heat shock protein *β*1 (HSPB1), which sequesters aggregated preproteins and facilitates their transfer to the proteasome.

Cytosolic ROS in conjunction with cytosolic precursor accumulation has also been implied in the activation of the mitochondrial unfolded protein response (UPR^mt^) (Sutandy et al., 2023), a pathway responding to protein misfolding in the mitochondrial matrix (Martinus et al., 1996; Zhao et al., 2002) (Figure 3a). Cytosolic precursors accumulate thereby as a consequence of an impairment of the matrix import motor that relies on mitochondrial HSP70 which instead acts on unfolded proteins in the matrix. Additionally, misfolded proteins inside mitochondria result in increased ROS generation at the respiratory chain, for example, by affecting turnover of damaged respiratory chain subunits. In the cytosol, these ROS oxidize the cytosolic protein DNAJA1 (Sutandy et al., 2023). Its oxidation leads to the enhanced recruitment of cytosolic HSP70 to accumulated precursors and the concomitant release of HSF1 from HSP70. Then, HSF1 is free to translocate to the nucleus and to activate transcription of mitochondrial unfolded protein response genes (Al‐Furoukh et al., 2015; Horibe & Hoogenraad, 2007; Zhu et al., 2021).

### 4.2 At the TOM complex

Partially or completely misfolded precursors (e.g., proteins that contain non‐native disulfide bonds) might also sterically block the TOM channel, thereby further contributing to the accumulation of precursors in the cytosol (Figure 3b). In yeast, blockage of the TOM channel has been shown to trigger response mechanisms like the mitochondrial protein translocation‐associated degradation (mitoTAD) pathway (Martensson et al., 2019) or the mitochondrial compromised protein import response (mitoCPR) (Weidberg & Amon, 2018) (Figure 3b). In the constitutively active surveillance pathway mitoTAD, the E3‐ubiquitin ligase Rsp5 ubiquitylates precursor proteins that arrest during translocation (Schulte et al., 2023). Subsequently, Ubx2, a dually localized component of the ER‐associated degradation machinery, recruits the cytosolic AAA‐ATPase Cdc48 to the TOM complex, facilitating the delivery of ubiquitylated precursor proteins to the proteasome (Martensson et al., 2019; Opalinski et al., 2018). Conversely, the mitoCPR is a stress‐induced mechanism that unclogs the TOM complex upon import stress (Weidberg & Amon, 2018). The mitoCPR involves the induced expression of the gene CIS1, which encodes a cytosolic protein that binds to mitochondria via Tom70 (Boos et al., 2019; Weidberg & Amon, 2018). Cis1 links the AAA‐ATPase Msp1 to Tom70, and Msp1 extracts arrested precursors for proteasomal degradation (Basch et al., 2020; Chen et al., 2014; Matsumoto et al., 2019; Okreglak & Walter, 2014; Weidberg & Amon, 2018). In mammalian cells, the outer membrane‐embedded E3 ubiquitin ligase MARCH5 interacts with the TOM channel and ubiquitylates several precursor proteins to induce their extraction and degradation (Ordureau et al., 2020; Phu et al., 2020). Whether the proteins contributing to these unclogging and stress response pathways are themselves redox‐sensitive has not been explored yet.

TOM channel clogging is also employed in a specific redox‐regulated fashion (Figure 3c). The protein FNIP1 contains redox‐sensitive cysteines. If present in a reduced state, they sensitize FNIP1 for ubiquitylation and subsequent proteasomal degradation. Upon high ROS levels, FNIP1 is present in the oxidized redox state allowing it to bind to TOM22, slowing down the import process. Conversely, upon low ROS levels, reduced FNIP1 can become ubiquitylated by the E3‐ubiquitin ligase CUL2‐FEM1B for its degradation. This frees the TOM22 receptor, allowing it to increase mitochondrial protein import and ensure the biogenesis, in particular of complex IV (Manford et al., 2020, 2021; McMinimy et al., 2024).

TOM channel components are also sensitive to oxidative modifications, and this has been shown to alter precursor recognition and binding, thereby modulating import selectivity under stress conditions (Fita‐Torro et al., 2025; Gornicka et al., 2014). Such redox‐sensitive tuning may allow cells to transiently restrict mitochondrial import during acute oxidative stress, limiting the accumulation of misfolded proteins within the organelle. This happens at the expense of precursor accumulation in the cytosol, which might be better tolerated due to better degradation capacities of cytosolic quality control machineries. For example, TOMM40 and TOMM70 can become modified at multiple cysteines (Mnatsakanyan et al., 2019). Moreover, the receptor subunit TOMM20 becomes degraded by the proteasome under oxidative stress; however, the underlying mechanism remains unclear (Wright et al., 2001). TOMM20 is also involved in a ROS‐dependent pyroptosis‐induction pathway (Fu et al., 2020; Xiao et al., 2020; Zhou et al., 2018). Increased ROS can cause the oxidation and oligomerization of TOMM20. Then, the proapoptotic protein Bax is recruited to mitochondria by oxidized TOMM20, which facilitates cytochrome *c* release to the cytosol to activate caspase‐3, eventually triggering pyroptotic cell death.

### 4.3 Inside mitochondria

Oxidative stress can decrease the mitochondrial membrane potential primarily by targeting and damaging IMM‐lipids and membrane proteins such as respiratory chain subunits, thereby decreasing transmembrane proton transport and increasing proton leak, and triggering the opening of the mitochondrial permeability transition pore (Choksi et al., 2004; Gorospe et al., 2023; Guo et al., 2013; Iqbal & Hood, 2014; Kirkinezos et al., 2005; Rottenberg, 2023; Satoh et al., 1997; Schopfer et al., 2009; Sousa et al., 2025). This reduction causes decreased protein import rates, or the arrest of protein precursors in the import channels.

Redox modifications of individual import translocase components could also directly influence translocation although it currently remains unclear when and to which extent oxidative modifications take place and how they impact mitochondrial protein import. Several translocase subunits contain conserved cysteine residues that are susceptible to reversible oxidation. This includes TIMM50 (Bleier et al., 2015; Xiao et al., 2020), TIMM44 (Xiao et al., 2020), and SAM50 (Xiao et al., 2020). TIMM50 is thereby also a nice example for a generator‐site specific modification as it becomes only modified from ROS released by complex III towards the IMS but not from the ones released by complex I (Bleier et al., 2015).

Oxidative stress also directly impairs mitochondrial protein import into the matrix by targeting the HSP70‐based import motor (Allu et al., 2015; Karri et al., 2019) (Figure 3d). Susceptible to oxidation is yeast Mge1 (GrpEL1 in mammals), the nucleotide exchange factor that regulates the ATPase cycle of mitochondrial Hsp70 and drives protein translocation through the TIM23 complex. Under oxidative stress, specific methionine residues in Mge1 undergo reversible oxidation, inducing structural changes that weaken its interaction with Hsp70 and reduce nucleotide exchange. This transiently slows ATP‐dependent matrix protein import. The mitochondrial methionine sulfoxide reductase Mxr2 counteracts this methionine oxidation and re‐establishes productive interaction with Hsp70. This redox control mechanism is evolutionarily conserved (Allu et al., 2018; Kisty et al., 2023). In mammals, oxidation of methionine 146 in GrpEL1 similarly disrupts HSP70 activity and mitochondrial protein homeostasis and is specifically reversed by R‐type methionine sulfoxide reductases (Allu et al., 2018).

Interestingly, yeast Tim17 (Badrie et al., 2025; Ramesh et al., 2016) and Tim22 (Okamoto et al., 2014; Wrobel et al., 2013; Wrobel et al., 2016) the central translocating subunits of the TIM23 and TIM22 pathways, respectively, contain disulfide bonds. These disulfides are likely introduced by a recently identified additional machinery for disulfide bond formation in IMM proteins, carried by the IMM protein Dbi1 (also Dmo2 or, in mammalian cells, DMAC1) (Badrie et al., 2025). The disulfide bond in Tim17 stabilizes the TIM23 complex. If absent, Tim17 shows decreased steady state levels, and import of MTS‐containing proteins is slowed. Conversely, the disulfide bond in Tim22 seems to be important for assembly of the TIM22 carrier translocase. If the disulfide bond is absent, Tim22 fails to assemble into the mature TIM22 complex, and carrier protein import is strongly impaired. The disulfide bonds, as well as the machinery, appear to be conserved in their human counterparts (Wrobel et al., 2016), but the mechanism is not yet validated in humans.

Disulfide bond formation can also occur in the mature part of precursor proteins destined for import into the mitochondrial matrix and is compatible with their translocation through the TIM23 complex. This has been demonstrated for proteins such as the yeast ribosomal protein Mrp10, its human counterpart CHCHD1, and the human complex I assembly factor NDUFAF8, which possess unusually weak MTSs and are, at the same time, substrates of the mitochondrial disulfide relay in the IMS (Longen et al., 2014; Peker et al., 2023). In these cases, disulfide bonds are introduced within the IMS prior to IMM translocation, resulting in the passage of partially folded, disulfide‐bonded helix–loop–helix motifs across the IMM.

### 4.4 A dedicated redox‐gated import pathway—the mitochondrial disulfide relay

**FIGURE 4:**
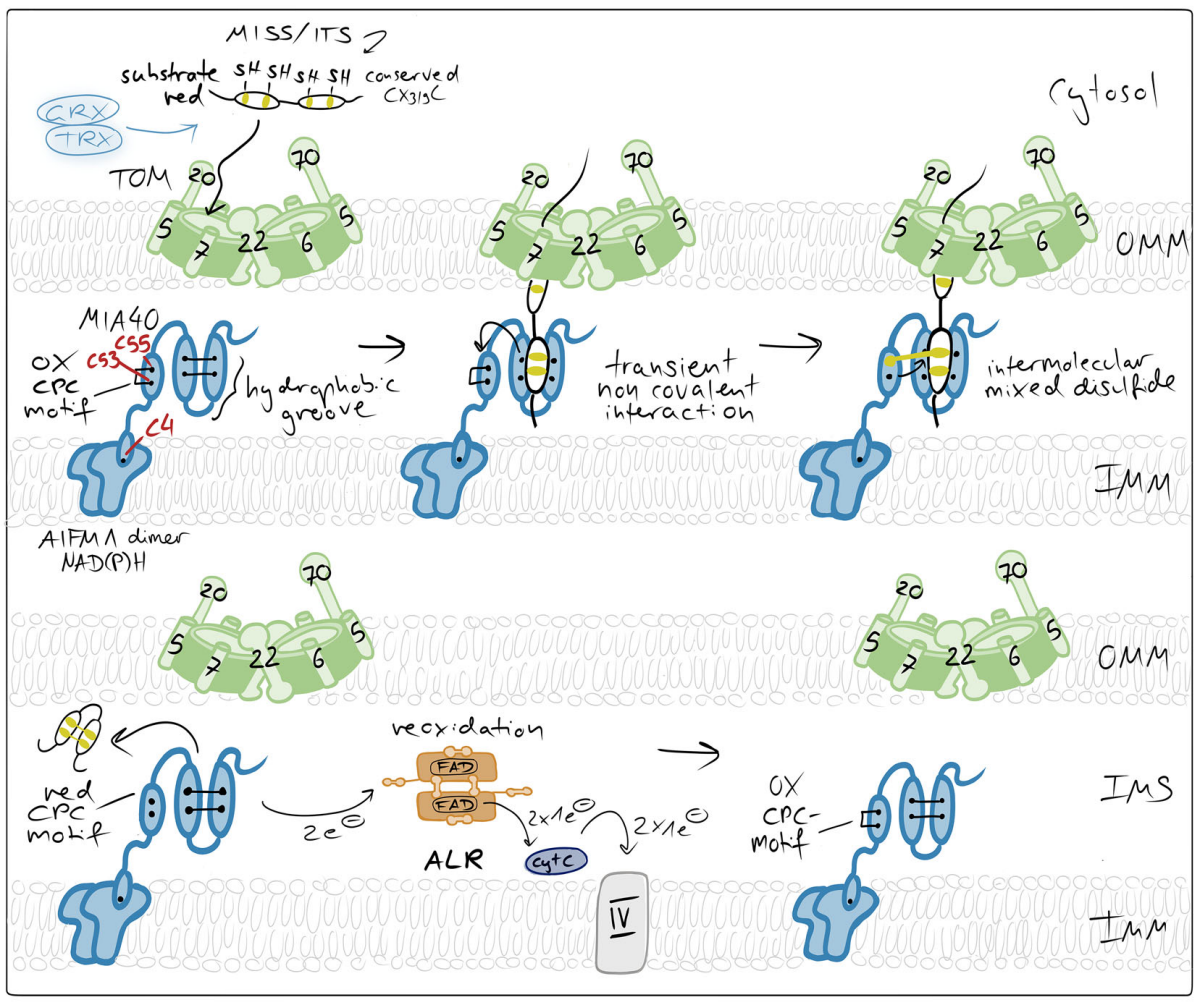
The mitochondrial disulfide relay. Incoming reduced and unfolded cysteine‐containing precursor proteins are imported through the TOM complex and oxidized by MIA40, which after an initial hydrophobic interaction links itself via an intermolecular mixed disulfide bond to the substrate, and thereby introduces intramolecular disulfide bonds. MIA40 thereby drives vectorial protein import and traps substrates in the IMS. MIA40 is reoxidized by the flavin‐containing sulfhydryl oxidase ALR, which transfers electrons to cytochrome *c* and ultimately to complex IV of the respiratory chain. In mammalian cells, AIFM1 serves two functions as an essential redox partner of MIA40: It attaches MIA40 to the inner membrane (IMM) and it activates the oxidoreductase.

The clearest example of redox regulation in mitochondrial protein import is the IMS disulfide relay system (Backes & Herrmann, 2017; Dickson‐Murray et al., 2021; Stojanovski et al., 2012; Zarges & Riemer, 2024) (Figure 4). This pathway mediates the import and oxidative folding of a distinct class of nuclear‐encoded proteins characterized by conserved cysteine motifs (twin‐CX_*n*_C as targeting motifs instead of N‐terminal MTS) and mostly compact helix–loop–helix folds. Unlike matrix import pathways that rely on ATP hydrolysis and chaperone‐assisted folding, the disulfide relay uses redox chemistry as its primary driving force, directly coupling protein import to oxidative folding.

The cysteine‐rich substrates of the disulfide relay pathway are synthesized in the cytosol and posttranslationally imported. They are maintained in their reduced state by the thioredoxin and glutaredoxin systems (Banci et al., 2013; Durigon et al., 2012; Morgan et al., 2009). Whether modification of cysteines prevents translocation across the OMM is unclear, but a significant share of these precursors is degraded *en route* to mitochondria (Bragoszewski et al., 2013; Finger et al., 2020; Lapacz et al., 2025) presumably to ensure that only import‐competent proteins become imported. At the OMM, at least some MIA40 substrates are recognized by TOMM20 (Marada et al., 2024).

Upon entry into the IMS, protein precursors encounter the import receptor and oxidoreductase MIA40 (in mammals also termed CHCHD4) (Figure 4). MIA40 is membrane‐anchored in yeast and a soluble protein anchored to the IMM in mammals via its interaction with AIFM1. Its defining structural feature is a conserved helix–loop–helix fold stabilized by structural disulfide bonds and exposing a redox‐active CPC motif (Banci et al., 2009; Kawano et al., 2009). The CPC motif is positioned at the tip of a hydrophobic substrate‐binding cleft, enabling simultaneous recognition and oxidation of incoming substrates (Sideris et al., 2009). Substrate proteins contain characteristic IMS targeting signals (ITS or MISS motifs), which form amphipathic helices that dock into this hydrophobic pocket, precisely aligning substrate cysteines for thiol–disulfide exchange (Koch & Schmid, 2014a; Milenkovic et al., 2009; Sideris et al., 2009).

The core mechanistic step of the disulfide relay is the formation of a transient semi‐stable intermolecular disulfide bond between MIA40 and the incoming substrate (Banci et al., 2009; Fischer et al., 2013; Habich et al., 2019; Koch & Schmid, 2014b; Mesecke et al., 2005; Peleh et al., 2016) (Figure 4). This happens through a nucleophilic attack of a cysteine thiolate from the substrate on the C‐terminal cysteine (C55) of the oxidized CPC motif of MIA40. The mixed disulfide intermediate enables subsequent thiol–disulfide exchange reactions that introduce intramolecular disulfide bonds within the substrate protein. These disulfides stabilize the folded conformation of the substrate and trap the protein in the IMS, preventing retrotranslocation through the TOM complex and thereby driving vectorial import without ATP consumption (Habich et al., 2019).

Following substrate oxidation, MIA40 is left in a reduced state and must be reoxidized to sustain continuous import. This function is performed by the sulfhydryl oxidase ALR; (Erv1 in yeast) (Allen et al., 2005; Bihlmaier et al., 2007; Mesecke et al., 2005). Structurally, ALR is a homodimeric FAD‐dependent oxidase with a four‐helix bundle architecture (Bien et al., 2010; Daithankar et al., 2010; Kay et al., 2006; Wu et al., 2003). Each monomer contains two redox‐active cysteine pairs, of which one is positioned adjacent to the FAD cofactor and one is present in a MIA40‐interacting flexible arm. ALR accepts electrons from reduced MIA40 via thiol–disulfide exchange and transfers them through its FAD cofactor to cytochrome *c* and ultimately to the respiratory chain, linking IMS protein import directly to mitochondrial electron transport (Allen et al., 2005; Bihlmaier et al., 2007; Peker et al., 2021). Importantly, this link positions the disulfide relay as an integral component of mitochondrial metabolism rather than an isolated folding module.

**FIGURE 5:**
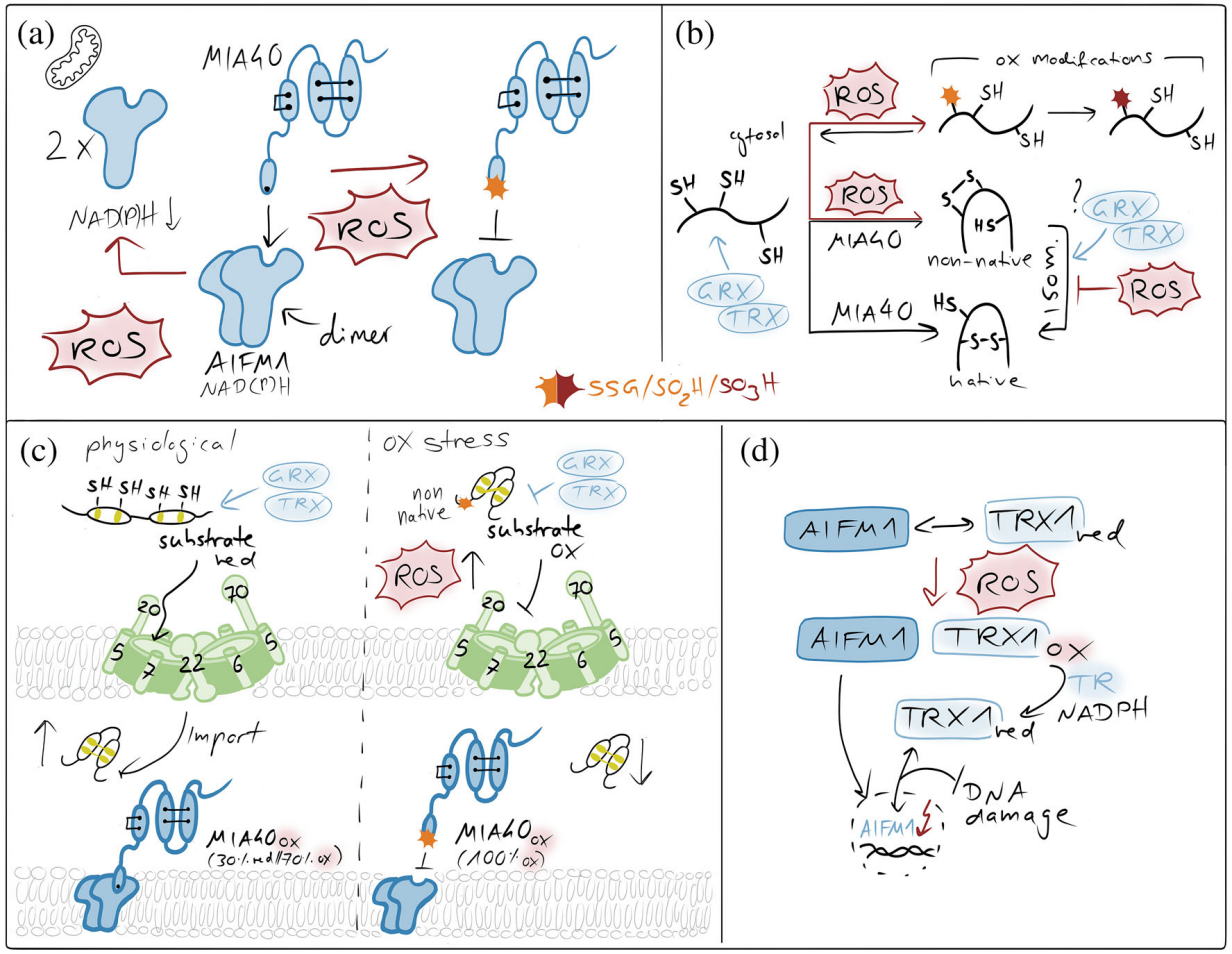
Redox imbalances affecting the mitochondrial disulfide relay. (a) ROS in affecting MIA40‐AIFM1 complex formation. NAD(P)H‐dependent AIFM1 dimerization is a prerequisite for the formation of the AIFM1‐MIA40 complex. Elevated ROS affect cytosolic NADH and NADPH levels and thereby lower dimerization efficiency. Moreover, ROS oxidize the redox‐sensitive cysteine 4 of MIA40 that is close to the AIFM1 interaction site in MIA40, thereby weakening MIA40's interaction with AIFM1 and lowering the activity of the disulfide relay. (b) ROS in preventing proper substrate oxidation. ROS can modify free cysteine thiols, for example, in unfolded disulfide relay precursors. These modifications can prevent disulfide formation. Moreover, ROS might also introduce non‐native disulfides in substrates which would require isomerization. Since isomerization relies on the presence of reduced cysteines, excessive ROS might also suppress this process. (c) ROS in influencing MIA40 and substrate redox states in intact cells. In intact cells, only 70% of the entire MIA40 pool is oxidized. This might be important for functions of MIA40 in target isomerization or reduction. ROS fully oxidizes MIA40, inhibiting those functions. Moreover, excessive ROS can also result in premature oxidation of substrates in the cytosol, preventing their import. Conversely, excess glutaredoxin (GRX) in the IMS has been shown to reduce disulfide bonds in newly imported substrates and in MIA40, thereby inhibiting productive oxidative folding. (d) Thioredoxin in maintaining AIFM1 functional in the IMS. In the IMS, TRX1 interacts with AIFM1 in a redox‐dependent manner that requires its active‐site cysteines. Oxidative stress disrupts this interaction, coinciding with AIFM1 nuclear translocation, while recovery restores binding over time. In the nucleus, reduced TRX1 limits AIFM1‐DNA interaction, thereby attenuating AIFM1‐mediated DNA damage and cell death (Shelar et al., 2015).

The activity of the mitochondrial disulfide relay is tightly controlled by the redox environment of the IMS (Figure 5). Efficient substrate oxidation requires sufficient oxidizing power to drive disulfide bond formation but also adequate availability of downstream electron acceptors to enable continuous recycling of ALR. Accordingly, perturbations of respiratory chain activity and cytochrome *c* availability directly modulate disulfide relay function. Inhibition of complex III has been shown to promote relay activity, whereas inhibition of complex IV impairs substrate oxidation and import, underscoring the dependence of the system on intact electron flow to the respiratory chain (Bien et al., 2010; Bihlmaier et al., 2007; Fischer et al., 2013; Kojer et al., 2012; Kojer et al., 2015; Mesecke et al., 2005). In addition to respiratory control, the redox buffering capacity of the IMS critically influences relay efficiency. Protection of the transient mixed disulfide intermediate between MIA40 and its substrates requires that glutaredoxin activity in the IMS remains low, thereby limiting excessive coupling of the relay to the highly reducing glutathione pool. Consistently, overexpression of glutaredoxins within the IMS delays disulfide relay‐mediated protein import by prematurely reducing catalytic disulfide intermediates in humans and yeast (Habich et al., 2019; Kojer et al., 2015). Finally, the functional organization of the disulfide relay depends on the interaction between MIA40 and apoptosis‐inducing factor mitochondria‐associated 1 (AIFM1) (Brosey et al., 2025; Hangen et al., 2015; Petrungaro et al., 2015; Rothemann et al., 2025; Salscheider et al., 2022). Binding of MIA40 to AIFM1 is essential for its activation and proper positioning within the IMS. This interaction is sensitive to cellular redox metabolism, as it depends on cytosolic NADH availability, with low NADH levels preventing AIFM1‐MIA40 complex formation. Moreover, reversible oxidative modifications of a cysteine residue in MIA40 (C4), located near the AIFM1 interaction interface, have been reported to occur under oxidative stress and may further modulate this interaction (Erdogan et al., 2018; Herrmann & Riemer, 2021).

Recent work has revealed that the substrate spectrum of the mitochondrial disulfide relay extends well beyond proteins with a classical twin‐CX_*n*_C architecture. These non‐canonical substrates—including adenylate kinase 2 (AK2), MICU1 and MICU2, FAM136A, ATP23, and CCDC127—often contain single disulfide bonds, non‐symmetrical or non‐consecutive cysteine spacing, or larger folded domains (Finger et al., 2020; Petrungaro et al., 2015; Weckbecker et al., 2012; Zarges et al., 2025). Despite this structural diversity, these proteins engage the disulfide relay through conserved mechanistic principles. They interact with MIA40 via hydrophobic IMS targeting signals, enabling transient mixed disulfide formation through the CPC motif and subsequent oxidative maturation. For several non‐canonical substrates, disulfide formation does not enforce a compact helix–loop–helix fold but instead fulfills regulatory or stabilizing functions. In the case of AK2, a central enzyme in adenine nucleotide homeostasis, disulfide bond formation stabilizes the protein against proteolytic degradation and promotes its functional retention within the IMS (Finger et al., 2020). For MICU1 and MICU2, oxidation induces and stabilizes a covalent heterodimer, providing a redox‐dependent mechanism for regulating mitochondrial calcium uptake (Petrungaro et al., 2015). Beyond proteins with exclusive IMS localization, a subset of non‐canonical substrates relies on the disulfide relay to achieve partial IMS import and dual cellular localization, frequently in a stress‐dependent manner. This has been reported for the copper chaperone of superoxide dismutase 1 (Ccs1) in yeast (Gross et al., 2011; Kloppel et al., 2010; Kloppel et al., 2011; Varabyova et al., 2013), as well as for anamorsin (Banci et al., 2011), p. 53 (Zhuang et al., 2013), apurinic/apyrimidinic endonuclease 1 (APE1) (Barchiesi et al., 2015), and cyclophilin D (CypD) (Equisoain Redin et al., 2025). In these contexts, the disulfide relay acts less as a dedicated folding machine and more as a redox‐dependent quality control checkpoint that regulates protein stability, localization, and function in response to cellular redox conditions.

Importantly, oxidation of non‐canonical substrates is often more sensitive to the redox state of the IMS than that of classical twin‐CX_*n*_C proteins. For example, the disulfide bond in AK2 appears less stable than those formed in canonical MIA40 substrates, rendering its maturation particularly dependent on sustained relay activity (Finger et al., 2020). Similarly, the intermolecular disulfide bond between MICU1 and MICU2 is formed relatively late during maturation, after both proteins have already been fully imported into the IMS (Petrungaro et al., 2015). Moreover, the spatial arrangement of cysteine residues in some non‐canonical substrates, such as Atp23 and AK2 (Finger et al., 2020; Weckbecker et al., 2012), raises the possibility that disulfide bond isomerization reactions are required during maturation. However, MIA40 itself displays only limited isomerase activity in vitro (Hudson & Thorpe, 2015; Koch & Schmid, 2014c), leaving the mechanism of disulfide rearrangement in mitochondria unresolved. In other oxidative folding environments, such as the endoplasmic reticulum and the bacterial periplasm, dedicated isomerases of the thioredoxin‐family fulfill this role. By analogy, the IMS may harbor yet unidentified isomerase activities, or local glutaredoxins and thioredoxins may facilitate iterative reduction and re‐oxidation cycles to enable correct disulfide pairing.

### 4.5 Outlook

Mitochondrial protein import is influenced by redox signals through energetic control, reversible oxidation of translocase components, regulation of oxidative folding pathways, and modulation of precursor stability. Excessive ROS can thereby compromise import and drive pathology, while signaling amounts can enable adaptive remodeling of the mitochondrial proteome. This remodeling is further driven by distinct mitochondrial protease machineries that themselves are subject to redox regulation. Understanding how these redox‐dependent mechanisms are integrated across compartments remains a central challenge.

## AUTHOR CONTRIBUTIONS

**Torsten Ochsenreiter:** Writing – review and editing; conceptualization. **Lidwina Hasberg:** Writing – review and editing; visualization; conceptualization. **Jan Riemer:** Writing – original draft; writing – review and editing; visualization; project administration; funding acquisition; supervision; conceptualization. **Viktoria Katharina Lauterbach:** Writing – review and editing; visualization; conceptualization.

## CONFLICT OF INTEREST STATEMENT

The authors have nothing to disclose and no conflict of interest.

## Data Availability

Data sharing not applicable to this article as no datasets were generated or analysed during the current study.
